# The Transient Mechanics of Muscle Require Only a Single Force-Producing Cross-Bridge State and a 100 Å Working Stroke

**DOI:** 10.3390/biology9120475

**Published:** 2020-12-16

**Authors:** Carlo Knupp, John M. Squire

**Affiliations:** 1 School of Optometry and Vision Sciences, Cardiff University, Cardiff CF10 3NB, UK; 2 Muscle Contraction Group, School of Physiology, Pharmacology & Neuroscience, University of Bristol, Bristol BS8 1TD, UK; j.squire@imperial.ac.uk; 3 Department of Metabolism, Digestion and Reproduction, Faculty of Medicine, Imperial College, London SW7 2BZ, UK

**Keywords:** muscle transients, myosin cross-bridge cycle, isotonic shortening, length steps

## Abstract

**Simple Summary:**

With modern increased computational power, newly developed computer programs can be used to simulate how muscle contracts. Here, we created, *in silico*, a “virtual” muscle that includes modelled myosin cross-bridges, and, using statistical mechanical methods, we calculated the macroscopic response of the muscle during contraction and as a result of applied transients. Good fits to many experimental observations were obtained with this simple model with one attached force-producing state and using a single cross-bridge step size of 100 Å.

**Abstract:**

An informative probe of myosin cross-bridge behaviour in active muscle is a mechanical transient experiment where, for example, a fully active muscle initially held at constant length is suddenly shortened to a new fixed length, providing a force transient, or has its load suddenly reduced, providing a length transient. We describe the simplest cross-bridge mechanical cycle we could find to model these transients. We show using the statistical mechanics of 50,000 cross-bridges that a simple cycle with two actin-attached cross-bridge states, one producing no force and the other producing force, will explain much of what has been observed experimentally, and we discuss the implications of this modelling for our understanding of how muscle works. We show that this same simple model will explain, reasonably well, the isotonic mechanical and X-ray transients under different loads observed by Reconditi et al. (2004, Nature 428, 578) and that there is no need to invoke different cross-bridge step sizes under these different conditions; a step size of 100 Å works well for all loads. We do not claim that this model provides a total mechanical explanation of how muscle works. However, we do suggest that only if there are other observations that cannot be explained by this simple model should something more complicated be considered.

## 1. Introduction

**Figure 1:**
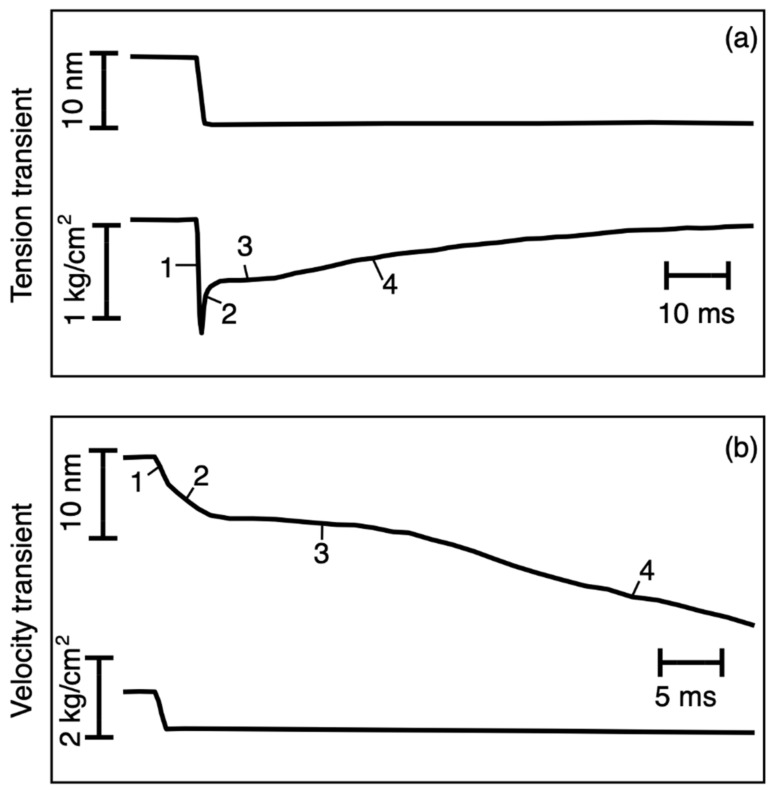
Illustration of transient experiments on muscle starting from the plateau of an isometric tetanus. Upper panel: length-step experiments showing the length trace (top) and the resulting tension transient (bottom). Lower panel: force step experiments where the load was suddenly reduced (bottom trace) and there was a length transient (top). Phase 1 is the change during the step, phase 2 is the rapid recovery in (**a**) or rapid shortening in (**b**), phase 3 is a slowing stage, and phase 4 is the gradual return to plateau tension in (**a**) or steady shortening in (**b**). Based on Huxley and Simmons (1971).

Studies of the mechanics of the cross-bridge cycle in muscle have often involved the application of rapid mechanical interventions to active muscle fibres. Huxley and Simmons [1] and Ford et al. [2] applied rapid shortening and lengthening steps to isometrically contracting fibres and obtained tension responses such as those in Figure 1a. With a shortening step, there was an instantaneous reduction in tension from the maximum T_o_ to the so-called T_1_ value. This was followed by a slower recovery of tension, the T_2_ curve, which oscillated as it approached a new steady isometric tension. Others (e.g., [3,4,5,6,7]) followed the length response of fibres contracting isometrically but then shortening after their load (the external force applied to the muscle) was suddenly reduced to a new constant value (Figure 1b). The muscle shortening velocity changed in a sinuous way until steady shortening was achieved. Related phases (1 to 4) can be seen in (a) and (b).

**Figure 2:**
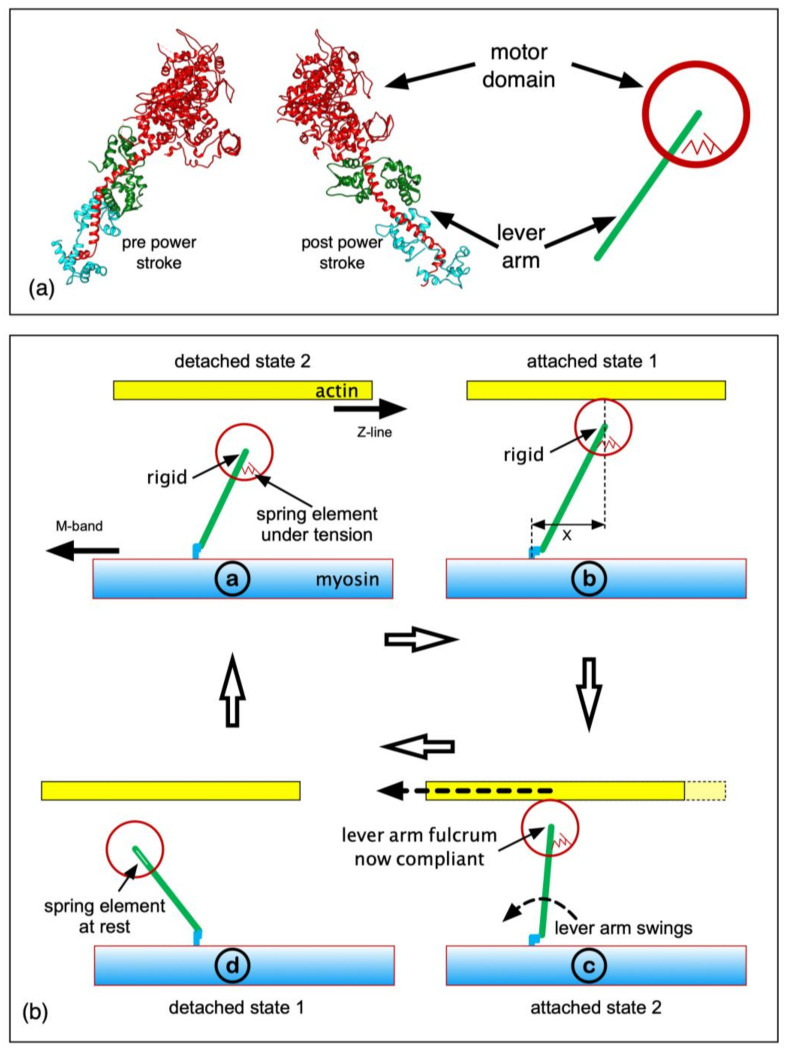
(**a**) The myosin head, showing the motor domain, lever arm and light chains in the putative pre-powerstroke state (left; Dominguez et al. [23]) and post-powerstroke state (middle; Rayment et al. [8]) assuming a horizontal actin (Z-band to the right). (**a**) Right: our approximation of a myosin head as a motor domain and lever arm, with a spring between the two that can pull back when stretched. (**b**) The simple mechanical cross-bridge cycle used in the simulations. For details, see text.

The force-producing myosin cross-bridges ([8]; Figure 2a) have an actin-binding ATPase motor domain providing energy for force generation, coupled through a converter to the lever arm, a long single α-helix around which two light chains wrap. The cross-bridge cycle on actin is thought to involve at least four steps [9]. ATP bound to the motor domain (M) is hydrolysed to ADP and inorganic phosphate (Pi), which remain bound (M.ADP.Pi). In activated muscle, the M.ADP.Pi complex binds weakly to actin in a rapid equilibrium state (A)M.ADP.Pi, which progresses to a pre-powerstroke strong state possibly associated with Pi release (AM.ADP.Pi or AM.ADP). After Pi release, ADP is released, giving the AM state (rigor-like). During the process AM.ADP.Pi > AM.ADP > AM, the cross-bridges exert force. AM heads bind another ATP and come off actin (M.ATP), M.ATP is hydrolysed to M.ADP.Pi, and the cycle repeats. The lever arm angles can change depending on the bound ligand (e.g., ATP, ADP.Pi, ADP or AM or their analogues) [10,11]. Force production and movement are thought to involve a rotation of the lever arm on a motor domain that is relatively rigidly attached to actin, although the motor domain changes its shape in the process.

We showed [12] that most of the instantaneous T_1_ change in length-step experiments is from the myosin and actin filament backbones and not the cross-bridges. However, presumably, the slower T_2_ curves must be due to the recovery responses of the actin-attached cross-bridges. Huxley and Simmons [1] argued, before the myosin head structure was determined, that this recovery could involve transitions between differently angled states of the myosin heads attached to actin. We would now say that the lever arm rotates on a relatively fixed motor domain on actin.

Here, we have modelled the transient responses in Figure 1 using a simple mechanical system, for a range of step sizes and timings in the tetanus. We wanted to determine the minimum mechanical system that would explain the observed transients. The model contained four mechanical states in the active cycle (Figure 2b): detached heads (detached state 2; DS2), strong binding heads producing no force (attached state 1; AS1), strong binding heads producing force causing the lever arm to swing if it is free to do so (attached state 2; AS2) and detached state 1 (DS1). In the Discussion, we consider the biochemical states that these might correspond to. Only if this simple mechanical model is not able to explain the mechanics results such as those in Figure 1 will we be forced to go to something more complex with more mechanical steps in the active cycle.

Modelling has been attempted previously (e.g., [1,13,14,15,16,17,18,19,20,21,22]), and, combined with X-ray diffraction results, Reconditi et al. [6] claimed that the head step size in load-step experiments depends on the load. We show that our simple four-state model can account well for the length and load-step results, without the need to postulate different lever arm step sizes, and that this relatively simple system explains results, including the X-ray diffraction observations, more closely than has been achieved before.

## 2. Materials and Methods

We used the mechanical cycle shown in Figure 2b. The transitions between structural states were represented as the probabilities that transitions will take place in time Δt = 0.02 ms (Table 1).

The model comprised a hexagonal array of ~170 myosin filaments (~50,000 myosin heads), with twice as many actin filaments. Because, even with modern computers, there is a limit to processing speed (we used parallel computations on seven processors), to reduce the number of free parameters, all the filament backbones were assumed to be non-compliant and were rigidly connected either to the M-band (thick filaments) or the Z-band (thin filaments), which were also non-compliant. We asked: “How well can the classic observations be explained by a simple mechanical model?”.

Each myosin filament had 50 crowns each of 3 head pairs, 145.7 Å apart axially. Each head had a force-producing internal spring that could be in one of two states: an unstretched, no-tension state (attached state 1, AS1) and a force-producing state (attached state 2, AS2), where the internal spring was stretched to a length determined by the separation (x) of the cross-bridge origin on myosin and its binding site on actin, plus a “step size”. See Table 1 for a list of parameters, along with their values as used in this paper.

**Figure 3:**
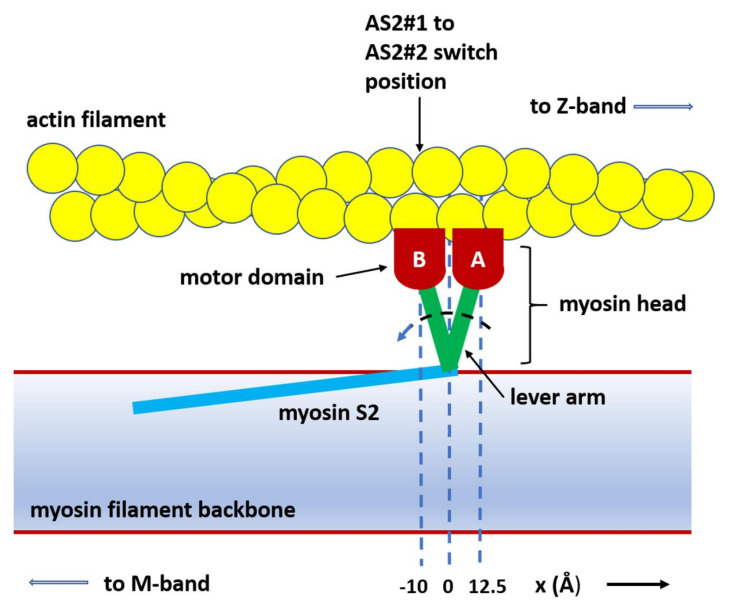
Illustration of the model with (**A**) the centre of attached state 1 (AS1) 12.5 Å in the positive ***x*** direction (away from the M-band) from the head origin on the myosin filament and (**B**) the motor position at the AS2#1-to-AS2#2 switch site at −10 Å.

If x is the axial displacement of the cross-bridge attachment to actin from its origin on myosin (Figure 2b and Figure 3), the force F exerted by the cross-bridge in state AS2 is:

F = k (x + (step size))(1)

with k being the spring constant (“cross-bridge stiffness” parameter; Table 1) of the internal spring. “x” can be negative EITHER if the initial head attachment on actin is towards the M-band relative to its origin on myosin OR if the thin filament with the attached head slides sufficiently past the thick filament towards -x without detaching. The attachment positions were chosen from a normal distribution set by two parameters: the “cross-bridge attachment offset” (the axial position of the centre of the distribution on actin) where the positive direction is towards the Z-band and the “cross-bridge attachment spread” (the standard deviation σ of the attachment Gaussian). The initially attached state (AS1) did not produce tension.

The cross-bridge internal spring was modelled as linking the myosin backbone (myosin S2, Figure 2) and the myosin rod (distal end of the cross-bridge lever arm). The step size therefore corresponds to the full lever-arm swing distance distal to the motor domain and is a limiting case for the internal spring. However, the spring is probably localized within the cross-bridge itself (as shown in Figure 2b); its length change would be smaller than the distal-end lever-arm movement, and its spring constant, greater, so that the product (step size × stiffness) is the same as the limiting case. The resulting tension effects would be the same.

The average half-sarcomere tension (the force exerted by the myosin heads) was calculated as:

(2) $$\mathrm{Tension}\;=\;\sum_{i}^{Na}k\left\{x_{i}+\left(\mathrm{step}\;\mathrm{size}\right)\right\}\;\frac{300}{N}$$

where *i* runs over all the AS2 heads (*Na*), *N* is the total number of attached cross-bridges, 300 is the number of cross-bridges per half myosin filament, *k* and the step size are as defined above and *x_i_* is the axial displacement of the *i*th cross-bridge. (Strictly, this number is 294 heads per half myosin filament [24]—49 crowns of 6 myosin heads each, with a missing crown near the filament tip—but we ignored this complication here.)

The calculations during the simulation were performed at regular intervals (Δt; Table 1). The time for any event is the sum of all the time intervals prior to that event.

We wanted to model the whole tension response from the initial time zero stimulus, so an extra “inactive” state was added to the four-state active cycle (Figure 2b), to model the start of the tetanus. It was not involved in the rest of the modelling. Thus, we have five states: the inactive state, detached states 1 and 2, and attached states 1 and 2. In relaxed muscle, all the cross-bridges were in the inactive state. At each interval Δt, the head state could change from the relaxed state to DS2, governed by the probability of cross-bridge activation (Table 1); then, once in this state, the state of each cross-bridge could change in the following ways:

From DS2 to AS1, governed by “probability of transition from DS2 to AS1’”.

From AS2 to DS1, governed by “probability of transition from AS2 to DS1”.

From DS1 to DS2, governed by “probability of transition from DS1 to DS2”.

The AS1-to-AS2 state change for force-producing heads was governed by (i) the AS1-to-AS2#1 probability parameter, if the axial displacement of the AS1 heads (*x*) was more towards positive *x* than the “AS2#1-to-AS2#2 switch length” (Figure 3), and (ii) AS1-to-AS2#2 probability parameter if the attached head was more towards negative ***x*** than the “AS2#1-to-AS2#2 switch length”. In other words, there is only one force-producing state (AS2), but the probability of reaching it depends on the value of ***x***.

The cross-bridges acted independently and state changes could occur at any time, but the cycle was constrained to proceeding in the forward direction, and, after activation, cross-bridges could only follow the sequence:

→ DS2 → AS1 → AS2 → DS1 → DS2 → etc.

The total sarcomere tension was the sum of the forces from all the cross-bridges in AS2.

If the actin filaments were free to slide, their positions were calculated in two steps:

(1) Calculate the actin filament velocity as (total cross-bridge tension on actins + load)/µ (where µ is the “viscous damping coefficient”);
(2) Calculate the actin filament movement as (actin filament velocity) * Δt.

The assumption was that the resultant force on the actin filaments was negligible. The µ value is in Table 1. The length steps were modelled as an instantaneous axial shift of all the actin filaments towards the M-band. From the positions and states of all the detached and attached cross-bridges, the whole A-band density profile was calculated and the meridian of the X-ray diffraction pattern computed (see [24,25,26]). The cross-bridges were treated as delta functions at either their origin on myosin for the DS1 and DS2 heads or their position on actin for the AS1 and AS2 heads. In this latter case, the position was shifted axially by a constant value (“centre of mass shift of attached cross-bridges”—see Table 1). Detached cross-bridges were convoluted with a Gaussian, centred at the cross-bridge origin, with a width of “sigma detached cross-bridges” and area of one. Different weights were assigned to heads in different states (Table 1). The bare zone length was 2 × the “half bare zone” parameter. The A-band profiles included 16 Gaussian functions, spaced axially by 3 × 145.7 Å, representing possible myosin filament backbone densities using the “centre of mass shift of extra Gaussians with 430 Å periodicity”, “sigma of extra Gaussians with 430 Å periodicity” and “weight of extra Gaussians with 430 Å periodicity” parameters.

Profiles created at different times during the cycle were fast-Fourier transformed, and the computed M3 (145.7 Å) peaks were analysed. The M3 peak contained two sub-peaks due to interference between the two half A-bands of each sarcomere (see [24] pp. 359, 360). The peak areas were calculated, and their sum (I_M3_) and ratio (R_M3_) plotted. Attached and detached cross-bridge populations were tracked and were plotted as functions of time or sarcomere length change.

## 3. Results

### 3.1. Length-Step Transients

**Figure 4:**
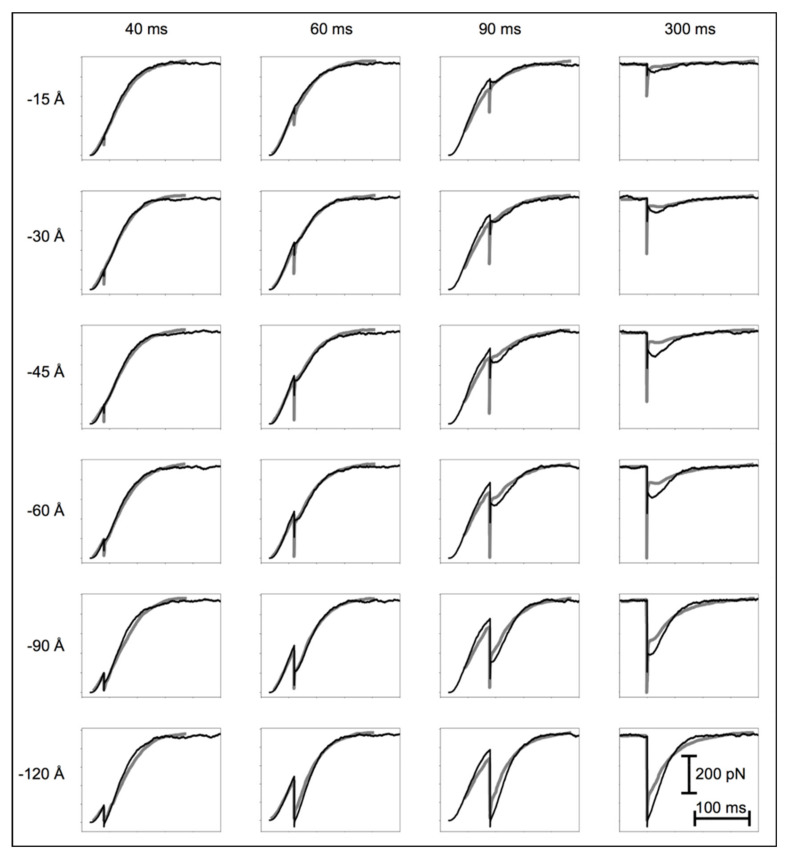
Experimental observations (grey traces) in length-step experiments of Ford et al. [2] and our simulations (black traces) of the same experiments using the cycle in Figure 2b and starting from the initial stimulus at zero time. The agreement over the whole set of experiments is reasonably good.

All the length-step experiments by Ford et al. ([2]—their Figure 1) were modelled (our Figure 4). First, we modelled the tetanus rising phase from time zero, without imposing shortening steps. All the experimental curves in Figure 4 include a latent period (~16 ms) before tension is recorded, due to the thin filament activation delay. The tension then increased gradually for about 10 ms. This was replicated by making the initial cross-bridge state inactive and gradually changing it to active. Without this extra step, there would be a sudden change in the gradient of the tension curve from its zero value, which was not seen experimentally.

The simulation then involved the four-state active cycle in Figure 2b. The model includes the minimum number of gross structural transitions through which the cross-bridges are likely to go. The changes in cross-bridge state were ordered so that a cross-bridge could either remain in the same state or change to the next later state around the cycle.

In our initial trial modelling, the probabilities for a state transition were constant, but this did not reproduce the plateau or inflection seen in the length response in the load-step experiments. We could replicate the plateau and other observations once the AS1-to-AS2 transition probability depended on the axial displacement of the attached cross-bridge (Figure 3). A satisfactory scheme was to define two different transition probabilities: one (AS1 to AS2#1) when the axial displacement of an AS1 head was at a higher positive *x* than a “switch” position, and the other (AS1 to AS2#2) when *x* was more towards negative *x* than this position.

To generate smooth curves, we carried out statistical analyses of the behaviour of 50,000 myosin heads, which took a great deal of computing time. For this reason, we were unable to set up a complete optimization algorithm (see [27,28]) to find the best parameter values. Instead, we carried out computational “experiments” to assess the effects of each parameter, identified preferred values, and then carried out local optimisation.

Figure 4 shows the experimental tension curves (grey) and simulations (black) after length steps at different times and of different sizes. Given the simplicity of the model, the correlation between the experiment and simulation is qualitatively good over the whole range of experiments. The main features not perfectly modelled are the instantaneous tension drop and the amplitude of the oscillations at later times in the tetanus. The discrepancies are likely due to the compliances of the thin and thick filaments, which, for lack of computing speed, we were unable to include in the modelling (we consider the possible effects of this in the Discussion). We have fully modelled the instantaneous tension drop elsewhere [12].

### 3.2. Isotonic Transients

**Figure 5:**
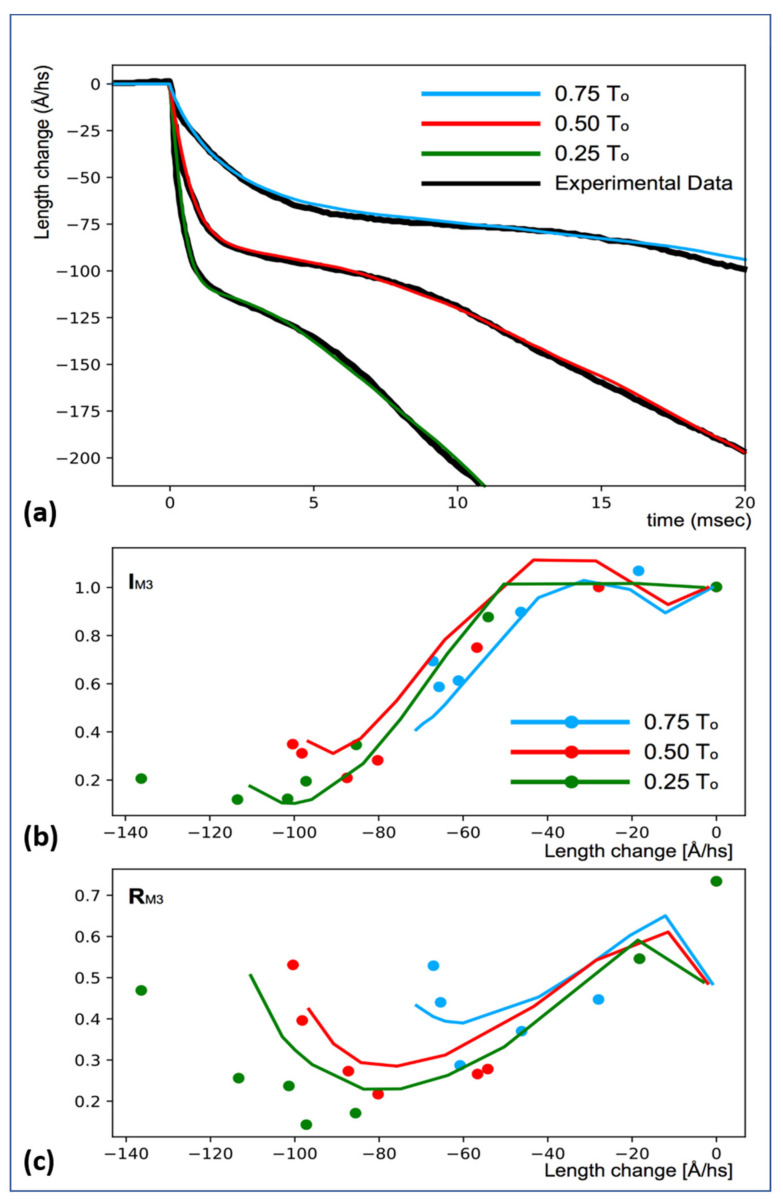
Results of modelling the isotonic transient studies of Reconditi et al. [6]. (**a**) The observed length changes as a function of load (black traces) and the modelled simulations in other colours (see key). (**b**) The computed X-ray diffraction intensities (I_M3_) for the traces in (**a**) against the observed values (dots). (**c**) Modelled R_M3_ values relative to the observed values (dots). For details, see text.

The model used for the length-step experiments (above) was used to explain the experiments by Reconditi et al. [6], where they measured the shortening time courses of an initially isometric muscle that shortened after a sudden reduction in load. Immediately apparent was that using the same values for the transition probabilities between states as used above led to very good shapes for the muscle-length-change curves, but on the wrong timescale. To obtain the right timescale, we had to change the values for the probabilities of the transitions between states. In addition, we needed different probabilities for different loads. With a 100% isometric load, all the probabilities of the transitions were the same as in the length-step simulations. After load reduction, the probabilities needed to be set instantaneously to new values (Table 1). All the other parameters remained unchanged. Figure 5a shows the experimental curves (black) and the simulations (coloured) with these new assumptions. The agreement is good.

In addition to length changes, Reconditi et al. [6] monitored the M3 (~145.7 Å spacing) X-ray meridional reflection. This reflection has two peaks due to interference between the two halves of the A-band. Figure 5b,c show the sum of these two M3 peaks (I_M3_) and their ratio (R_M3_), respectively, as a function of muscle shortening with experimental data (circles), and the continuous curves show our new simulations. The cross-bridge positions were those found to explain the muscle shortening curves. No attempt was made to tailor the results to fit the X-ray diffraction observations. In addition, we did not make use of the head crystal structure, or the mass and position of the lever arms. Their effects were explored elsewhere [29]; the lever arm probably contributes little to the observed M3 changes. Here, we were only interested in seeing how well the cross-bridge populations alone could explain the M3 interference data. The X-ray diffraction observations are quite well explained by our new, very simple model. The agreement is probably better than that in Reconditi et al. [6], and the conclusions are different; the step size throughout was 100 Å (Table 1).

### 3.3. Population of States: Length Steps

**Figure 6:**
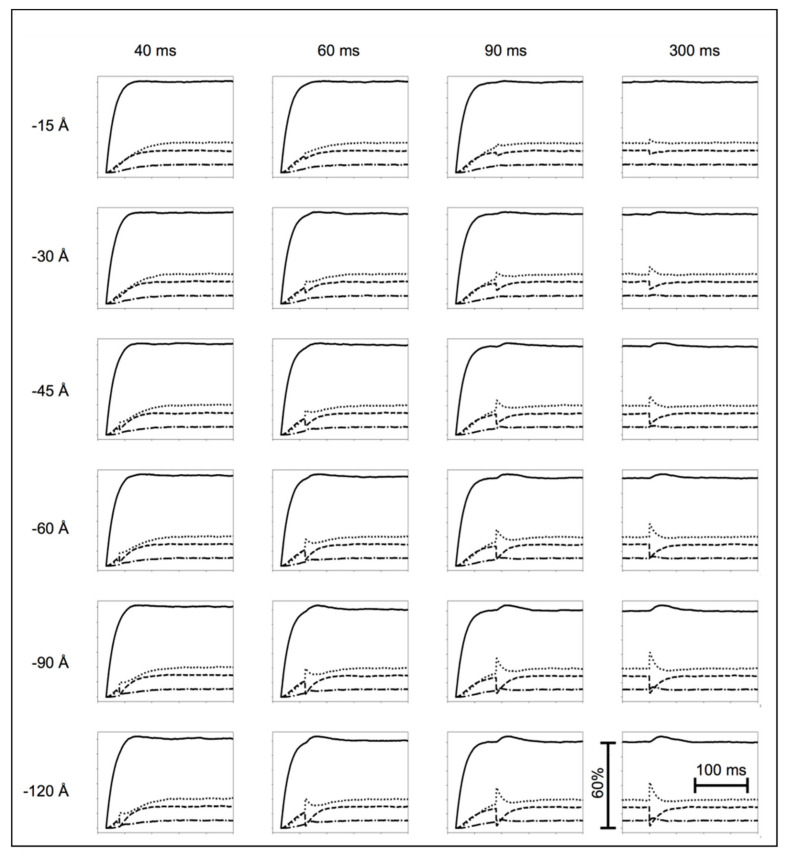
Variation of populations in the modelling of data in Figure 4. Detached state 2 population—continuous line; attached state 1 population—segmented line; attached state 2 force-producing population—dotted line; detached state 1 population—alternating segmented–dotted line. For details, see main text.

Figure 6 shows the varying populations of states over time for the length-step simulations (Figure 4). All the cross-bridges were “off” in the latent period, and then, all the populations increased, the rest population the most, as cross-bridges were drawn from the starting 100% “off” state. DS1 was the least populated. Ignoring the length-step effects, all the populations increased at an ever-slowing rate towards their steady-state, maximum-isometric-tension, values. The plateau populations were DS2 60%, AS1 15%, AS2 20% and DS1 5%. In the length steps, the AS1 population quickly dropped, accompanied by an increase in AS2 and a smaller increase in the population of DS1 and DS2. The population changes depended on the size of the step.

### 3.4. Population of States: Isotonic Shortening

**Figure 7:**
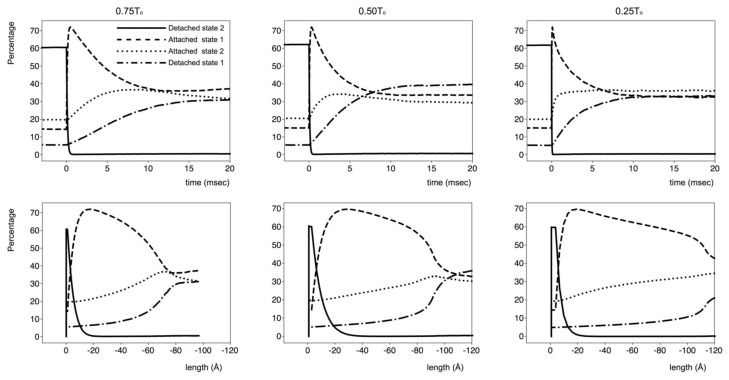
Populations of the various states in Figure 2b throughout the isotonic experiment modelling in Figure 5. Top panel, populations against time. Lower panel, populations against distance. Detached state 2 population—continuous line; attached state 1 population—segmented line; attached state 2 force-producing population—dotted line; detached state 1 population—alternating segmented-dotted line. For details, see main text.

Figure 7 (top panels) shows the cross-bridge populations for the simulations in Figure 5, for loads of 75% (left), 50% (middle) and 25% (right) of T_o_ plotted against time. The lower panel shows populations plotted against distance (time notionally still from left to right). The trends of the populations were very similar but were scaled depending on the load. After the load change and rapid early length drop, the DS2 population quickly decayed to a small value. The AS1 population increased to ~70% and then, after 10–20 ms, slowly decayed to a constant value, depending on the load. The AS2 population had a smaller initial increase (maximum 30–40%) and then decreased to a relatively constant 30% after 15 ms. The DS1 population increased to about 30–40%.

The DS2 population was small for most lengths (Figure 7; lower panels). The AS1 (no force) population peaked between 0 and −20 Å, decreased slowly at intermediate (−*x*) lengths and then dropped suddenly at lengths further towards the *-x* direction before reaching a constant value. The AS2 force-producing population increased steadily to reach a peak at lengths that corresponded to the sudden drop of the AS1 population. The DS1 population increased slowly at smaller lengths and then more rapidly, also in correspondence with the sudden drop in the AS1 population.

## 4. Discussion

We have shown that a relatively simple mechanical model of the muscle cross-bridge cycle can explain a host of experimental results in a reasonable way. There were many more observations than parameters to fit, so the problem was over-determined. Considering other possible models, we found that a simple three-state model in which the DS1 and DS2 populations were merged to one state would account well for the length-step experiments (Figure 4) but not the isotonic shortening experiments (Figure 5). Another possible model had three attached states, two of which (AS2 and AS3) were force producing, and there was a specific lever-arm position where the switch from AS2 to AS3 occurred (similar to the ideas in (1)), but with this model, we have not (to date) been able to find parameters that produce good results.

### 4.1. Isometric Length-Step Experiments

**Figure 8:**
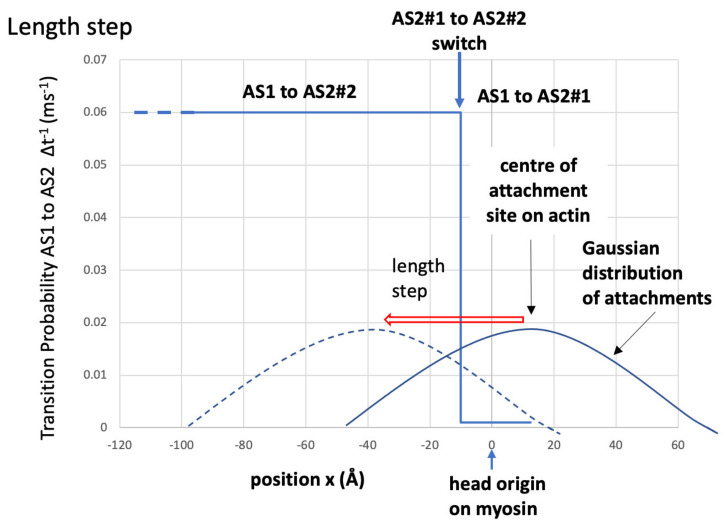
Illustration of the possible positions of heads attaching in AS1 (x = +12.5 Å) with a Gaussian spread (solid bell-shaped curve) as listed in Table 1, the probability change across the AS2#1-to-AS2#2 switch position (solid blue line), and the effect of a step-length change (red arrow) in bringing heads to the AS2#2 position (dashed bell-shaped curve) where the AS1-to-AS2 transition is more likely. (The heights of the bell-shaped curves are not to scale.)

We needed to introduce an inactive state to account for the latent period before the cross-bridges enter the main four-state active cycle. Calcium release from the sarcoplasmic reticulum and binding to troponin on the actin filaments, causing the steric blocking by tropomyosin to be removed, all takes time [30,31,32,33,34,35,36,37,38,39,40,41,42]. The latent period is the delay in the activation mechanism and is not something intrinsic to the cross-bridges themselves. The time course of the number of cross-bridges in AS2 closely mimics the time course of tension production by the muscle. Since tension is produced only by AS2 cross-bridges, it can be thought of as the product of the average force exerted by the AS2 heads and their number. If the AS1-to-AS2 transition probability did not depend on the attached head axial displacement (i.e., there was no switch), shortening the muscle would have no effect on the population of states, and the tension would drop only because the spring within each AS2 head would be shortened. Tension recovery would be due to the on-going cycling of cross-bridges. Since the rate of increase in the new cross-bridges in AS2 would be unchanged during sarcomere shortening, there would be an oscillation-free tension recovery with a time course similar to that of the tension build-up after the initial activation of the muscle. Some sort of transition within the AS2 population is essential (Figure 8).

There is a significant difference if the AS1-to-AS2 probability depends on the axial displacement of the AS1 cross-bridges. We chose the simplest possible dependency for this probability (Figure 3 and Figure 8) depending on the head position relative to the switch length. Since the cross-bridges are biased to attach to actin with positive displacement, the initial rate for muscle tension (because of the AS2#1 cross-bridge population growth) would be mostly dictated by the probability of transition from AS1 to AS2#1, which is the smaller of the two.

After a length change, the AS2 cross-bridge springs reduce and the tension drops. Concurrently, the attachment probability Gaussian (Figure 8) moves left to take many heads across the switch position to where a transition to AS2 is more likely. These cross-bridges quickly transition to AS2, providing a quick increase in force and total muscle tension. Here, the AS1 population is small, few cross-bridges can transition from AS1 to AS2, and there is a tension plateau. However, cross-bridges keep cycling, the AS1 population replenishes, and the AS2 population gets back to normal. The tension recovery is then like the increase at the start of the tetanus.

This mechanism is modulated by the number of cross-bridges in each state at the time and by the extent of the length change, which both influence the fraction of AS1-to-AS2#2 cross-bridges. Figure 6 shows the number of cross-bridges in each state as a function of time, from which it is possible to verify that the populations follow the trend discussed above.

### 4.2. Isotonic Muscle Contraction Experiments

**Figure 9:**
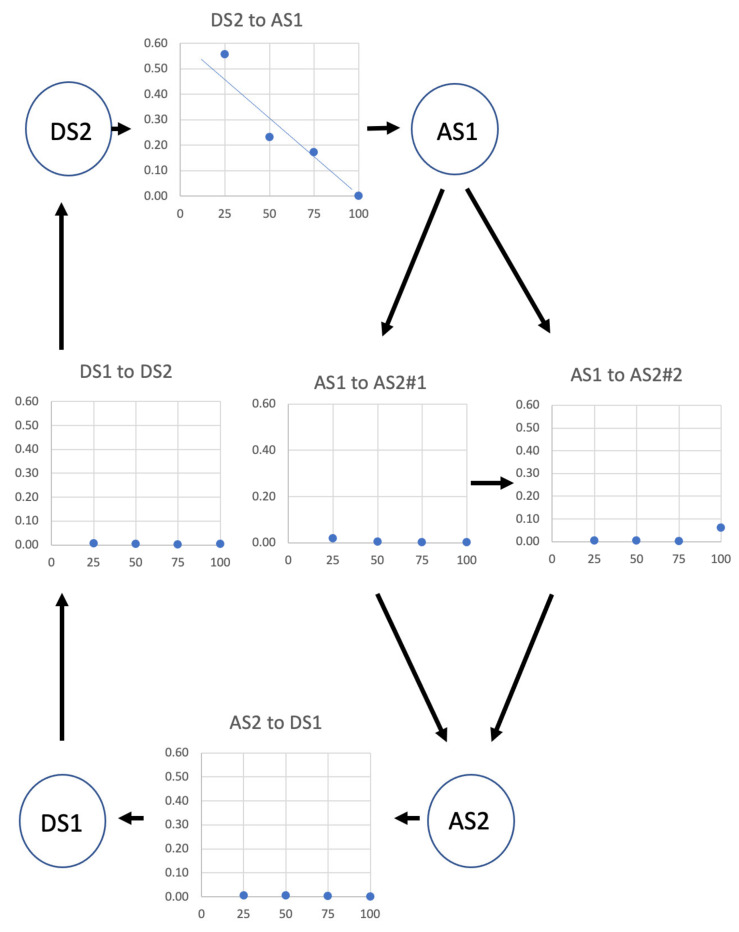
Variations in transition probability (boxes, vertical axis) around the cycle in Figure 2b as a function of load in the isotonic experiments in Figure 5 (horizontal axis). The biggest changes are in the DS2-to-AS1 transition, which increases almost linearly as the load reduces. For details, see text.

The same active muscle cycle explained the isotonic muscle contraction experiments of Reconditi et al. [6]. Initially, the muscle is in an isometric contraction, with the same parameter values as used in the isometric length-change experiments. After load reduction, all the parameter values were kept the same except for the state transition probabilities (Table 1). Figure 9 shows the variation in the transition probabilities around the cycle as a function of load (all plots on the same scale). The DS2-to-AS1 probability dominates and increases almost linearly with load reduction. This is not surprising. With the actin filaments sliding past the myosin filaments, new actin target areas [43,44,45,46] will be continuously presented to the myosin heads, and this will increase the probability of a cross-bridge transition from DS2 to AS1. The lower the load, the faster the actin filaments move and the more rapidly new sites will be presented. Thus, the DS2 population will be sharply diminished in favour of AS1 heads. If, at the same time, the other transition probabilities changed by a relatively smaller amount (Figure 9), the AS1 population increase would be followed by its decrease as the AS2 population increased, because of both the initially large number of cross-bridges now in AS1 and the greater number of AS1 heads being taken past the switch position to become AS2 force-producing heads. Eventually, there would be a decrease in the AS2 population and a rise in the DS1 population, until an equilibrium were reached (see Figure 8, upper panel).

During the isometric initial phase, the force exerted by the cross-bridges in AS2 is equal to the isometric tension. This tension, with a smaller contribution from newly transitioned cross-bridges, is what drives the rapid muscle contraction in the first few milliseconds after load reduction. The force from AS2 cross-bridges is pulling against the applied load, which influences the extent of muscle shortening at this early stage; the greater the load, the smaller the shortening. The initial shortening velocity depends on the AS2 population, which depends on the transition probabilities. After the initial rapid shortening, many AS2 cross-bridges are far away from their origin, their tension drops because of internal spring shortening, and there is a reduction in contraction velocity. Shortening then accelerates as new AS2 heads are recruited that exert a greater force. All populations then reach a steady state, the force exerted by the AS2 cross-bridges stabilises, and eventually, the muscle shortens at a constant velocity.

The state populations and their positions were used to calculate the changes in the M3 reflection during muscle contraction (Figure 5b,c). The experimental data are broadly well reproduced, even though there was no attempt to manipulate the transition probabilities to fit these data. There are obvious discrepancies at the initial stages, which may be the consequence of the abrupt way in which the transition probabilities were made to change after the initial isometric phase. There may also be a contribution from thick and thin filament compliances (see below). This may also explain the imperfect fit at high shortening lengths. However, trying to account for all these discrepancies would have made the model much more complicated, without adding to the fundamental insights derived from it.

### 4.3. Limitations of the Model

Because of the limits of present computational power, there was no serious attempt to use optimised global searching (e.g., by simulated annealing) for finding preferred values for the parameters. In addition, the thick and thin filament compliances were not considered (but see below), and structural information on the cross-bridge shape was not included. We are aware that isotonic experiments have been carried out over longer times than we have modelled (e.g., [7]), but the time constraints on modelling with so many (~50,000) cross-bridges put limits on what can be achieved in a reasonable time. Additionally, for the sake of simplicity, only a limited set of the possible transitions between states was allowed, with the transition between the states occurring instantaneously, which might not be the case. The model presented here is a simple “mechanical” model. The different mechanical/structural states of the cross-bridges need not correspond in a one-to-one manner to defined biochemical states, and a direct comparison between them is not trivial. For a direct comparison, and a reconciliation of the known biochemical rate constants, the different biochemical states need to be incorporated into the model, and each set of biochemical states needs to be associated with a corresponding mechanical one. In this paper, we have not yet considered the effects of applying step stretches to isometric muscles (e.g., [47,48]), and we have not considered the possible effects of temperature jumps (e.g., [49,50]). Another limitation is the fact that we did not include in the model a mechanism that reproduces the dependency of the cross-bridge rate constants on the muscle load; we just assumed that there is one. Nevertheless, we believe that the predictive powers of this simple model are remarkable and informative when thinking about the details of the molecular mechanism of muscle contraction, and they are open to testing.

### 4.4. The Possible Effects of Filament Compliance

In a previous publication [12], we considered the properties of the T_1_ curve as in Figure 1. We showed that this curve can be almost wholly explained by compliance in the myosin and actin filaments themselves, with little contribution from cross-bridge compliance [51,52]. We also showed that the value of the cross-bridge stiffness (1/compliance) must be much greater during the length step than had been determined before (we estimated about 0.8 pN/Å). In the present study, we were unable to include filament compliance in our calculations for lack of computing power, but we now consider what effect filament compliance might have on our results. We have already mentioned that the instantaneous tension drops in our length-step simulations in Figure 4 are not as great as observed, and this difference must be due to the absence of filament compliance.

The main novel idea in Knupp and Squire [12] is the suggestion that much of the instantaneous cross-bridge stiffness in length steps is due to weakly binding myosin heads [53]. Such heads are in a rapid equilibrium between the M.ADP.Pi detached state and the (A)M.ADP.Pi weakly attached state. The transitions between the two are so fast that in a normal muscle contraction on the several-millisecond time scale, these heads provide very little stiffness. However, if the muscle is stretched or shortens very rapidly (e.g., in less than 1 ms), many of these heads are trapped in the attached state during the length change and the muscle appears stiff. The stiffness increases either with an increased speed of length change or with an increasing population of weakly binding heads [51].

We suggested that in the weakly binding heads, the junction between the motor domain and lever arm might be quite rigid, hence the high stiffness of the heads, and that one of the effects of the initial strong binding of myosin to actin (possibly after Pi release) might be a loosening up of the cross-bridge so that its stiffness is only around 0.1 pN/Å and the head is released to act as a spring, providing a force over a full step size of around 100 Å. The same idea is built into Figure 2b. The preferred spring constant that explains the data in Figure 4 and Figure 5 is 0.07 pN/Å, close to the theoretical 0.1 pN/Å mentioned in Knupp and Squire [12].

To see what effect filament compliance might have on the length-step experiments, it is necessary to imagine myosin and actin filaments shortening during, for example, a 40 Å step, which reduces the measured tension to zero, providing an average shift for weakly attached heads between the site on myosin from which they come and the site on actin where they bind of around 12 Å (see [12]). At the same time, heads in a strong-force-producing state with low stiffness may change their shape on actin with a lever arm swing that also moves the distal end of the lever arm, on average, by 12 Å, thus lowering the force that they produce. In summary, a 40 Å shortening step will reduce the myosin and actin filament lengths, which lowers the tension, and at the same time, the lever arms of the average strong-force-producing attached heads will move by around 12 Å, also lowering the tension. The T_2_ recovery will have a mixture of heads shifting across our #1-to-#2 switch position, thus increasing the force-producing population and gradually increasing the force, during which some length changes in the actin and myosin filaments will also occur.

One of the features of the modelled length-step responses is that, sometimes, the oscillations in the T_2_ curves are slightly too great (e.g., Figure 4; 300 ms; −45, −60 and −90 Å). We think that the effect of the inclusion of filament compliance, making the filaments themselves locally more elastic, would be to tend to smooth out these responses. This would have the effect of smoothing tension oscillations so that the excursions are not as large as in Figure 4 and the modelled curves would better fit the observations. It should also be recognised that the experimental curves themselves are not entirely consistent. Even the shapes of the observed tension traces in the basic tetanic contractions (ignoring the steps: Figure 4) are not quite the same. We have used a single set of parameters to model all the experimental curves in Figure 4, and with experimental variation, some of these traces will inevitably fit better than others.

In the case of the isotonic contractions (Figure 5), once the load step has been applied, the force on the myosin and actin filaments will be constant, on average. During the load step itself, the filaments will instantaneously change length, depending on the load, and this might contribute to the very start of the length changes in Figure 5. During steady shortening, the load is constant, so compliance effects should be small. However, local variations in the stretch of the actin and myosin filaments during the steady-state shortening phase may also explain some of the discrepancies of the I_M3_ and R_M3_ curves, since the amount travelled by the cross-bridge at any given time may not coincide with the overall shortening of the muscle.

### 4.5. Comparison with Other Kinds of Result

**Figure 10:**
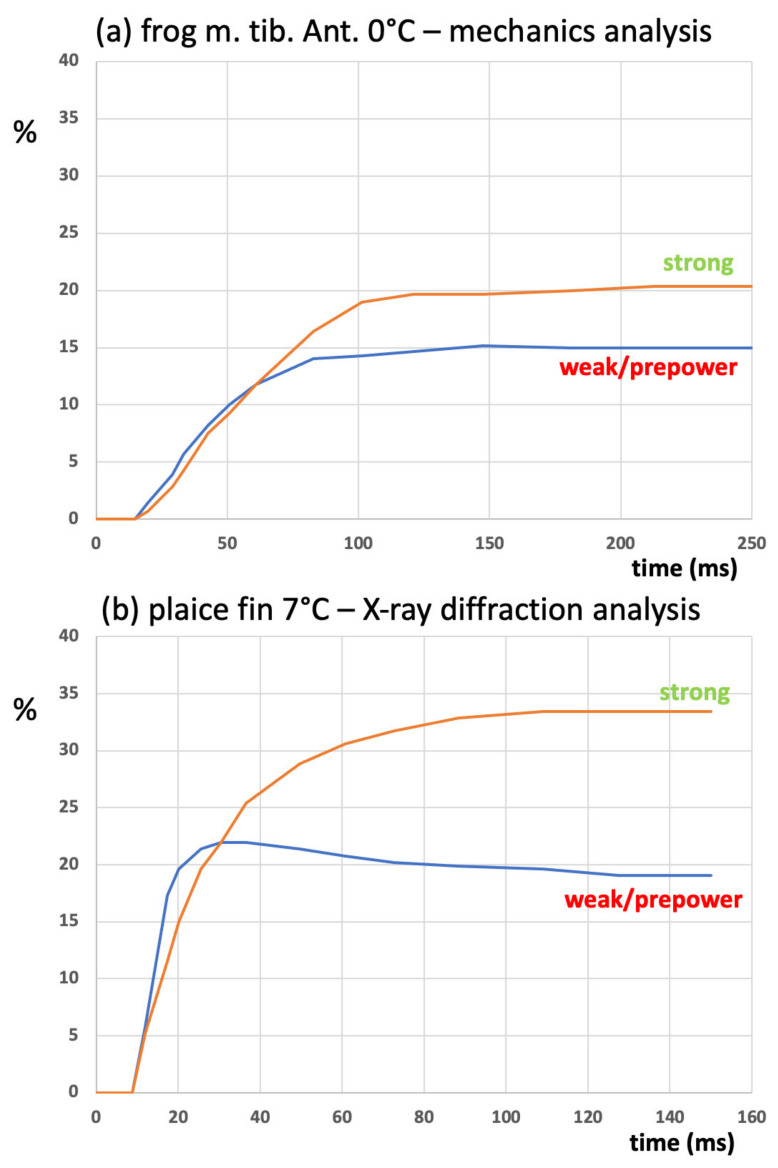
(**a**) The time courses of the population changes in strong-force-producing (AS2) heads and weak/pre-powerstroke heads (AS1) in the present modelling of an isometric tetanus in frog *m. tib. ant.* Muscle, and (**b**) the same parameters determined by fitting of time-resolved X-ray diffraction patterns from bony fish muscle [28].

In a previous paper on active bony fish muscle [28], we estimated the changing populations of heads in the weakly binding/first-attached state (AS1) and the strong-force-producing state (AS2) during the rising phase of tetanic contractions based solely on the changing intensities of the equatorial X-ray reflections from the A-band lattice. The estimates of the same parameters come from the present paper, but for frog *m. tib. ant.* (Figure 10a). Note, first, that the fish response (Figure 10b) is quicker than the frog response, so the plots are on different time scales. The fish muscle analysis shows the weak/pre-power population increasing ahead of the strong population and overshooting slightly before settling down to a steady 20% after 80 to 100 ms. The strong population increases steadily to around 34%.

The results in the present paper for frog muscle, based on a totally different experimental approach (mechanics, not X-rays), also show the weak/pre-power state (AS1) ahead of the strong (AS2) state, but this time, with a slower muscle, there is no overshoot. The two states plateau at about 20% for AS2 and 15% for AS1. In both cases, the variation of tension with time closely follows the population of the strong (AS2) heads.

That the responses in Figure 10 are slightly different is not surprising. They are from different animals and different muscle types and were recorded at different temperatures (frog, ~0 °C; fish, ~7 °C). They also have different A-band lattices; frog muscles have a superlattice; bony fish muscles, a simple lattice [54,55]. It is known that temperature has a marked effect on the tension produced, which may partly explain the different strong populations at the plateaus of the isometric tetani. In addition, the tension rise in the fish muscle is so fast that there is a build-up of the weakly binding/pre-power state that causes the overshoot before the force-producing state is reached. The slower frog muscle does not have such a build-up before tension is produced.

Despite these differences, similar trends can be seen in both sets of observations.

### 4.6. What Can Be Learned about the Cross-Bridge Cycle?

We can estimate the turnover rate (the number of cycles of each myosin head per second). For the isometric case, it is about 10 s^−1^. This increases almost linearly with the load reduction to about 110 per second at a load of 25% T_o_.

As discussed above, the different states in the cycle in Figure 2b are probably covering several identifiable structural and biochemical states (see Figure 19 of Houdusse and Sweeney [10]). DS2 is presumably a set of pre-powerstroke states: M^+^.ATP, M^+^.ADP.Pi and the detached part of weakly binding heads (A)M^+^.ADP Pi. AS1 is presumably the attached part of weakly binding (A)M^+^.ADP.Pi heads, together with a first, strongly attached, non-force-producing state AM*.ADP.Pi (where + and * denote different myosin head motor states). The AS2 state is either AM.ADP.Pi, AM.ADP and AM if Pi release follows the initial force generation or AM.ADP and AM if force production follows Pi release. DS1 will be M.ATP (the post-rigor state), and this will lead back, again, to the starting M^+^.ATP and M^+^.ADP.Pi structures.

We note that the fit here to the observations on muscle mechanics does not depend on:

(i) Detailed knowledge of the myosin head shape, apart from knowing there is a motor domain and a lever arm;
(ii) Knowledge of specific rate constants between the biochemical states around the cycle;
(iii) Knowledge of the energy wells for different states;
(iv) Postulating different power stroke lengths for different loads;
(v) Postulating that the behaviour of one head in a myosin molecule depends on the behaviour of the other head.

As discussed above, Figure 10 shows that the results presented here, and those from previous, totally independent, time-resolved X-ray diffraction studies of contracting bony fish muscle [28], are very similar. Both muscle mechanics and muscle equatorial X-ray diffraction can be modelled with just two attached states of the myosin heads on actin, one not producing force and the other producing force. The future modelling of other mechanics observations will surely add subtleties to the scheme outlined here, but the fact remains that many of the classic experimental observations of muscle behaviour do not need anything more complicated to describe what is happening. A simple cross-bridge cycle on actin with a lever arm swinging through 100 Å works very well.

## 5. Conclusions

We have tried to find the simplest possible mechanical system that can explain many of the published observations on muscle mechanical transients. This consists of a four state myosin head (crossbridge) cycle on actin in active muscle with one force-producing state and a myosin head working stroke of 100 Å. To explain the isotonic shortening results it was necessary to have the probability of transition of attached myosin heads from the non-force-producing (AS1) to the force producing (AS2) state a function of the attached head position relative to its origin on myosin as described by a single transition position (the switch position) beyond which the AS1 to AS2 transition became much more probable. We do not claim that this mechanical system is the last word on the mechanics of the myosin head cycle on actin, and further mechanics experiments may add to the mechanism described here. But we believe that it is desirable to fully explore what can be explained by a relatively simple crossbridge cycle such as the one presented here and only to make the cycle more complicated if other observations on muscle mechanics demand it.

## Figures and Tables

**Table 1:** Parameters used in the modelling and their values.

Parameters	Length Step Simulation	Length Change Simulation When T = 25% to	Length Change Simulation When T = 50% to	Length Change Simulation When T = 75% to	Length Change Simulation When T = 100% to
Δt (ms)	0.02	0.02	0.02	0.02	0.02
					
Probability of cross-bridge activation [Δt^−1^]	0.00080	0.00080	0.00080	0.00080	0.00080
Probability of transition from DS2 to AS1 [Δt^−1^]	0.00035	0.55678	0.23077	0.17251	0.00035
Probability of transition from AS1 to AS2 #1 [Δt^−1^]	0.00100	0.01893	0.00565	0.00143	0.00100
Probability of transition from AS1 to AS2 #2 [Δt^−1^]	0.06000	0.00381	0.00353	0.00285	0.06000
Probability of transition from AS2 to DS1 [Δt^−1^]	0.00107	0.00617	0.00498	0.00255	0.00107
Probability of transition from DS1 to DS2 [Δt^−1^]	0.00400	0.00681	0.00372	0.00257	0.00400
					
Step size [Å]	100.0	100.0	100.0	100.0	100.0
Cross-bridge stiffness [pN Å^−1^]	0.070	0.070	0.070	0.070	0.070
					
AS2 to DS1 cutoff length [Å]	−10.00	−10.00	−10.00	−10.00	−10.00
Cross-bridge attachment offset [Å]	12.50	12.50	12.50	12.50	12.50
Cross-bridge attachment spread (σ) [Å]	46.25	46.25	46.25	46.25	46.25
					
μ [pN ms Å^−1^]	n/a	2.27	2.27	2.27	2.27
					
Half bare zone (Å)	n/a	821.29	821.29	821.29	821.29
Centre of mass shift of attached heads (Å)	n/a	26.16	26.16	26.16	26.16
Weight of detached state 2 cross-bridges	n/a	0.68	0.68	0.68	0.68
Weight of attached state 1 cross-bridges	n/a	1.12	1.12	1.12	1.12
Weight of attached state 2 cross-bridges	n/a	0.950	0.950	0.950	0.950
Weight of detached state 1 cross-bridges	n/a	0.001	0.001	0.001	0.001
Sigma of detached cross-bridges (Å)	n/a	37.500	37.500	37.500	37.500
Centre of mass shift of extra Gaussians with 430 Å periodicity	n/a	5.544	5.544	5.544	5.544
Weight of extra Gaussians with 430 Å periodicity	n/a	1.218 × 10^6^	1.218 × 10^6^	1.218 × 10^6^	1.218 × 10^6^
Sigma of extra Gaussians with 430 Å periodicity	n/a	60.075	60.075	60.075	60.075
(For all length parameters, positive is towards the Z band.)					
